# Intraosseous ganglion cyst mimicking chondrosarcoma on MRI: a case report

**DOI:** 10.1186/s40001-022-00631-0

**Published:** 2022-01-13

**Authors:** Eun Hye Seo, Yu Sung Yoon, Jang Gyu Cha, Hee Kyung Kim

**Affiliations:** 1 Department of Radiology, Soonchunhyang University Bucheon Hospital, Soonchunhyang University College of Medicine, 170 Jomaru-ro, Bucheon, 14584 Republic of Korea; 2 Department of Pathology, Soonchunhyang University Bucheon Hospital, Soonchunhyang University College of Medicine, 170 Jomaru-ro, Bucheon, 14584 Republic of Korea

**Keywords:** Olecranon process, Intraosseous ganglia, Chondrosarcoma, Magnetic resonance imaging, Computed tomography

## Abstract

**Background:**

The intraosseous ganglia is a benign cyst, rarely locate in the olecranon process. As intraosseous ganglia can mimic malignant bone tumor, computed tomography (CT) is important for diagnosis even when magnetic resonance imaging (MRI) suggests malignant bone tumor, such as chondrosarcoma.

**Case presentation:**

In this paper, we report a 42-year-old woman with intraosseous ganglia in the olecranon process of the ulna. She complained pain in right elbow for 3 weeks. MRI revealed an intraosseous mass which initially diagnosed as chondrosarcoma. However, followed computed tomography (CT) demonstrated scattered intralesional gas and no underlying mineralization, and we can exclude chondrosarcoma from diagnosis.

**Conclusions:**

The intraosseous ganglia can mimic bone tumor in MRI; therefore, CT is essential for accurate characterization of bone tumor. Even if MR imaging strongly suggests chondrosarcoma of the bone, CT should be performed as additional study.

## Background

Intraosseous ganglia are benign cystic lesions consisting of fibrous tissue with extensive mucoid degeneration [1]. These cysts occur less frequently in the upper extremities and olecranon involvement is extremely rare [1, 2]. It can also cause pain [1–3].

We report a case of intraosseous ganglia in the olecranon process of the ulna, which was initially interpreted as chondrosarcoma with high signal intensity (SI) on T2-weighted fat-suppressed imaging, intermediate SI on T1-weighted imaging, and peripheral lobular enhancement. The patient also exhibited perilesional bone marrow edema, cortical breach, and extraosseous extension, which are common with malignant bone tumors [4]. However, a final radiologic diagnosis of intraosseous ganglia was reached based on CT before surgery, because scattered gas was observed within the presumed tumor, suggesting endogenous causes such as possession of cavity means hypocellularity and negative pressure due to juxtaarticular location. Its unusual location and MRI findings of intraosseous ganglia mimicking a chondrosarcoma were interesting and informative.

## Case presentation

A 42-year-old woman presented to the outpatient clinic with continuous pain in her right elbow that started 3 weeks prior. She had previously undergone conservative physical therapy at another hospital, but her symptoms were not relieved. The patient reported a Numeric Rating Scale (NRS-11) score of 2 [5]. She had no recent history of trauma or elbow injury, and had no complaints of fever, local heat, or pain in other joints. On physical examination, there was no tenderness, swelling, range of motion limitation, nor external wound on the right elbow. The patient had no other relevant medical history or abnormal laboratory findings.

**Fig. 1:**
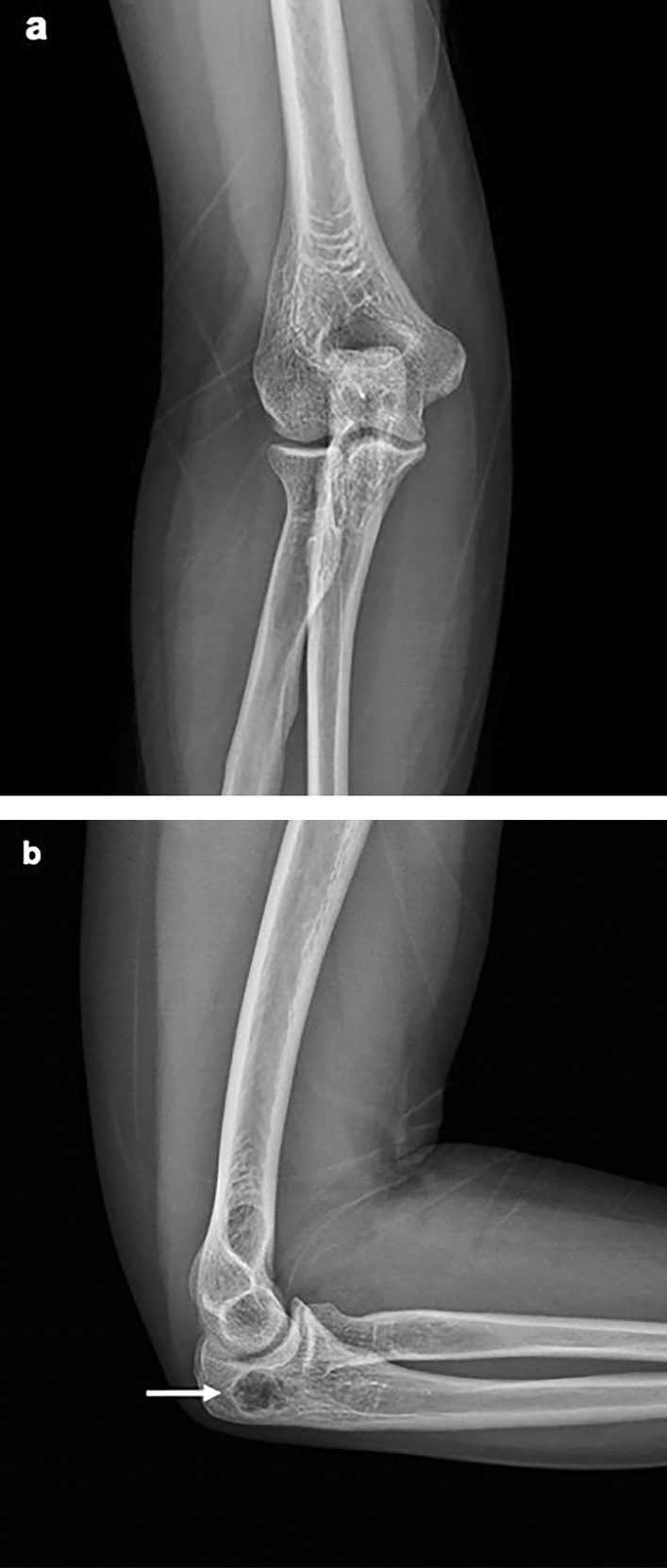
Right elbow radiograph (lateral projection). **a** In AP radiograph, it was difficult to find abnormality, because the olecranon process and the humerus overlapped. **b** A partially ill-defined osteolytic lesion ~ 2.0 cm in extent (white arrow) in the olecranon of the right ulna without discernible underlying mineralization

**Fig. 2:**
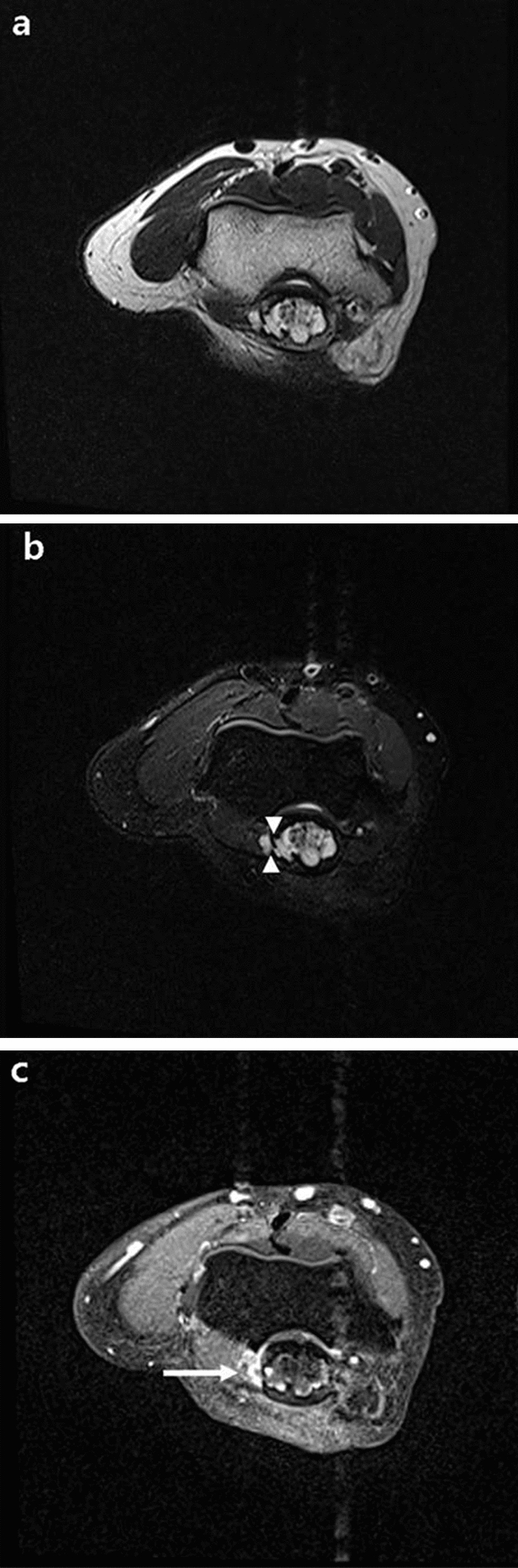
MRI of the right elbow. In-phase axial T2-weighted (T2W) (**a**), water only T2W (**b**), and T1-weighted (T1W) fat-suppressed contrast enhanced (**c**) images show a 1.5 × 1.3 × 2.3 cm sized intraosseous lesion with endosteal scalloping and peripheral enhancement. The lesion extended through cortical breach (white arrowheads) with enhancing extraosseous component (white arrow)

**Fig. 3:**
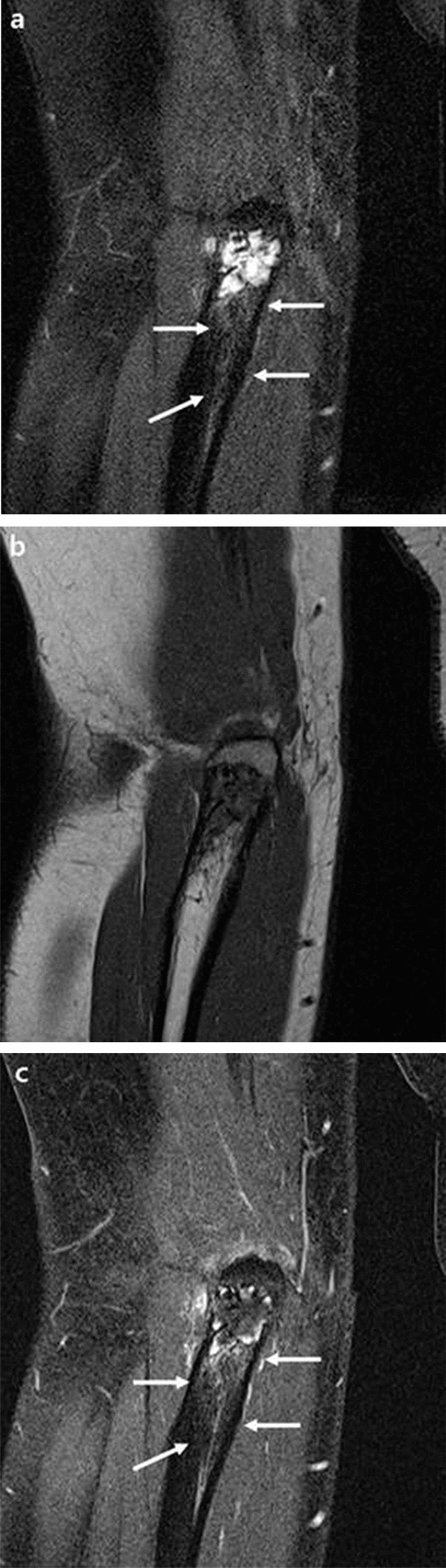
Coronal T2W fat-suppressed (**a**), T1W (**b**), and T1W fat-suppressed contrast-enhanced (**c**) images showed a mass with lobulated T2 high signal intensity containing a lobular enhancing periphery, which had subtle peritumoral bone marrow edema and enhancement (white arrows)

**Fig. 4:**
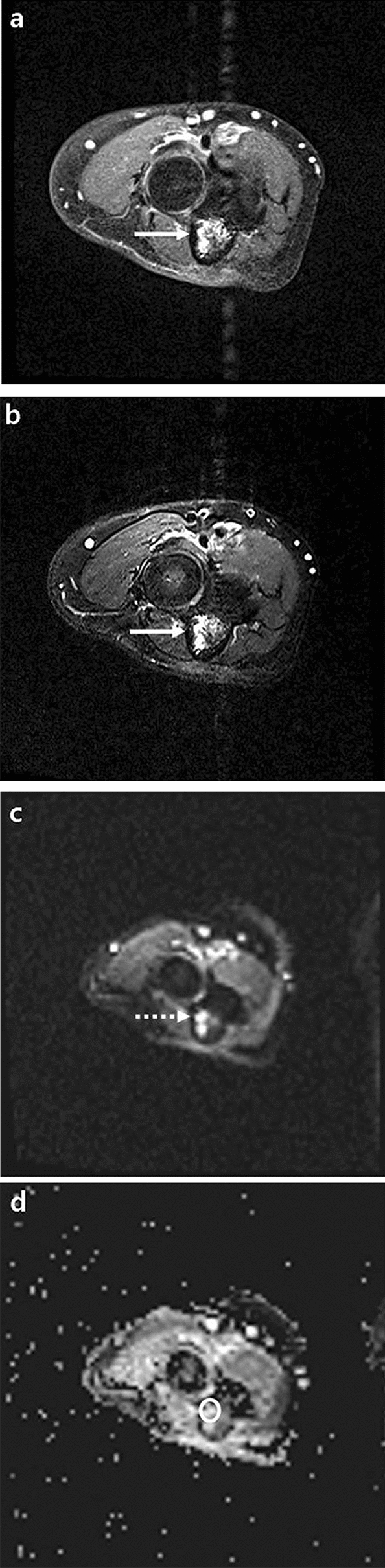
Axial T2W DIXON (**a**), T1W fat-suppressed contrast-enhanced (**b**), high *b* value (b = 1000) diffusion-weighted (**c**), and ADC map (**d**) images demonstrated an inferior-side-dominant enhancing solid portion, which showed diffusion restriction on high *b* value scan (average ADC value; 0.85, minimum ADC value; 0.62, maximum ADC value; 1.07)

Plain radiographs of her right elbow joint demonstrated an osteolytic bone lesion 2.0 cm in extent in the olecranon process. In AP plain radiograph, it was difficult to accurately detect the lesion, because the olecranon process and the humerus overlapped (Fig. 1a). In lateral projection, we found a geographic osteolytic lesion with partially ill-defined margin at distal portion (Fig. 1b). Contrast enhanced MRI was performed using a 3.0-T MRI scanner (Magnetom Skyra, Siemens, Germany). MRI revealed an approximately 1.5 × 1.3 × 2.3-cm-sized intraosseous mass with marginal lobulation, peripheral lobular enhancement with a regional enhancing portion, and prominent endosteal scalloping. In addition, the lesion had a focal cortical breach with extraosseous extension and perilesional bone marrow edema. There was no definite evidence of joint involvement (Figs. 2, 3, 4).

**Fig. 5:**
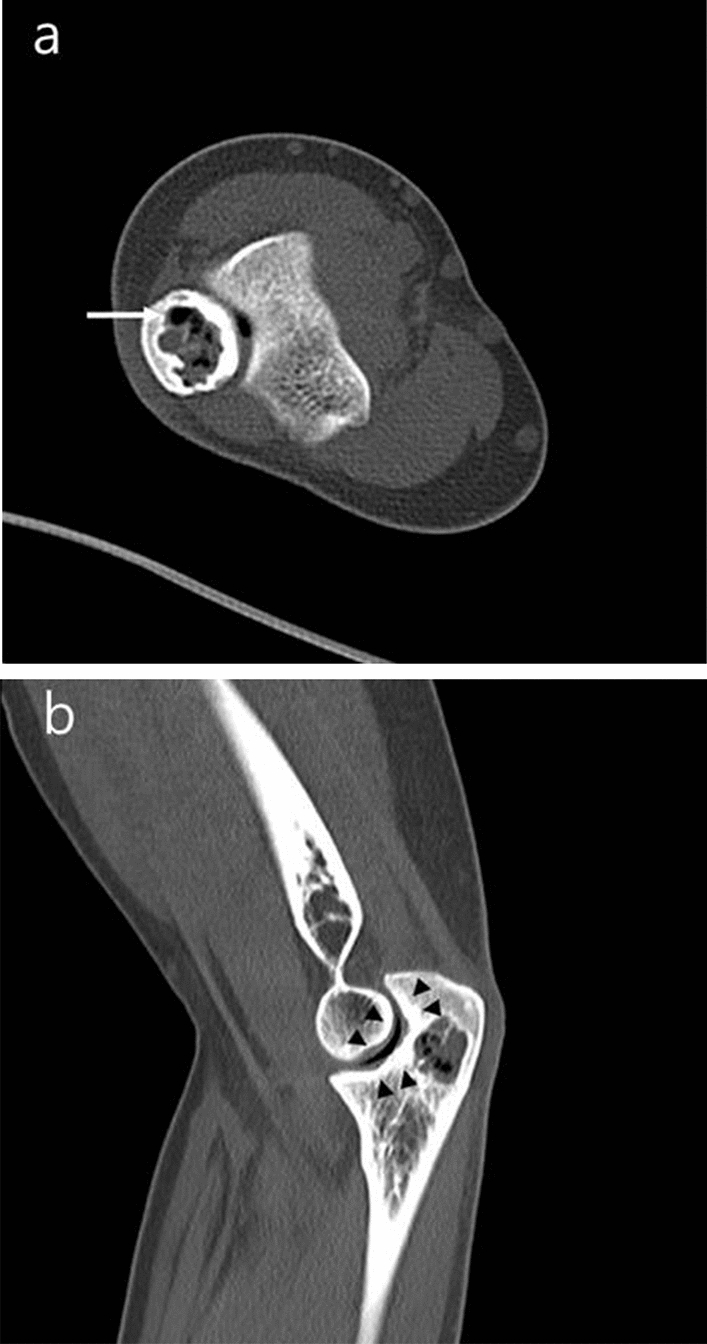
Axial (**a**) and sagittal (**b**) CT scan revealed intralesional gas (white arrow) without evidence of underlying osteoid or chondroid mineralization. Vacuum phenomenon was observed in ulnotrochlear joint with subcortical sclerosis and articular surface dimpling (black arrowheads), but there is no continuation between joint and osteolytic bone lesion

CT was performed to clarify the underlying mineralization. On CT, no underlying osteoid or chondroid mineralization was observed except thin septa suggesting hyperdense structure (Fig. 5).

**Fig. 6:**
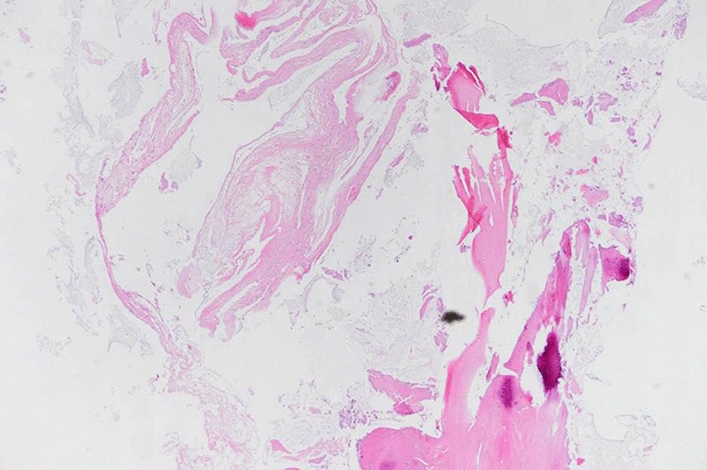
Microscopic findings of the specimen revealed fragments of fibrous membranous tissue with mucoid degeneration and no epithelial lining (hematoxylin and eosin, × 40)

The mass was surgically removed by curettage and elbow pain was relieved after surgery. Pathologic examination of the specimen revealed fragments of fibrous membranous tissue with mucoid degeneration and no epithelial lining (Fig. 6).

## Discussion

Ganglion cysts are fluid-filled lumps that typically develop in the soft tissue along tendons and joints [6, 7]. Intraosseous ganglia are rare and commonly located in the tibia, fibula, humerus, ulna, radius, carpal and tarsal bones, acetabulum, and scapular bone [1, 8–11]. The pathophysiology of intraosseous ganglia remains unclear, but it has been suggested that mechanical stress or trauma, synovial herniation, mucoid degeneration, and intramedullary metaplasia of mesenchymal cells may be the main causes [1, 12, 13].

Gas within the bone can be seen under various conditions, such as emphysematous osteomyelitis, pneumatocyst, osteonecrosis, and postoperative emphysema [14]. The gas results from gas-forming pathogens, exogenous air, and nitrogen gas from soft tissue due to distraction induced negative pressure [14, 15]. In negative-pressure related cases, some lesions have been reported in near completely normal joints without evidence of degenerative changes or a clear connection to the joint space [14]. Maldague et al. [16] believed that the presence of gas in a fracture was due to a lack of tissue, fluid and blood. Likewise, gas within bone tumors suggests a cellular paucity, indicating that reabsorption of gas to surrounding tissues does not occur easily or well liberating environment of nitrogen gas [1, 17]. In our case, a lack of tissue with mucoid content in the intraosseous ganglia may have contributed to the development of negative pressure, and the juxtaarticular location was also affected by negative pressure due to joint movement and maintenance. That negative pressure can result in a decrease in gas solubility. There was adjacent subcortical sclerosis and articular surface irregularities, but no clear connection was observed in CT and MRI. Gas collection was only identified on CT.

According to previous literature, gas in the vertebra or intervertebral discs, and formation of gas due to infection can be excluded if there is no evidence of concomitant infection, such as osteomyelitis or paravertebral soft tissue lesions [15]. To the best of our knowledge, no malignant bone tumors with air in the lesion have been reported in the English literature. It is believed that the high-density tissue of malignant tumors contributes to gas reabsorption. In our case, the reasons for a diagnosis of chondrosarcoma based on MRI were as follows: greater than two-thirds endosteal scalloping of the normal cortical thickness, a peripheral lobular enhancing pattern, cortical breach, and an enhancing solid extraosseous component. These findings strongly suggest chondrosarcoma [4]. Even on retrospective MRI review, air was not clearly defined. CT is optimal diagnostic method for intraosseous ganglia, because it can accurately illustrate underlying mineralization and even small amounts of gas [14, 18].

## Conclusion

A final radiologic diagnosis of intraosseous ganglion cyst was reached with CT before surgery based on scattered gas within the lesion. Intraosseous ganglion cysts can mimic bone tumors on MRI; therefore, CT is essential for accurate characterization of bone lesions and differentiation of intraosseous ganglia from chondrosarcoma.

## Data Availability

Not applicable.

## Abbreviations

CT: Computed tomography
MRI: Magnetic resonance imaging
SI: Signal intensity
